# Childhood Incident Asthma and Traffic-Related Air Pollution at Home and School

**DOI:** 10.1289/ehp.0901232

**Published:** 2010-04-06

**Authors:** Rob McConnell, Talat Islam, Ketan Shankardass, Michael Jerrett, Fred Lurmann, Frank Gilliland, Jim Gauderman, Ed Avol, Nino Künzli, Ling Yao, John Peters, Kiros Berhane

**Affiliations:** 1 University of Southern California, Los Angeles, California, USA; 2 St. Michael’s Hospital, Toronto, Ontario, Canada; 3 University of California, Berkeley, California, USA; 4 Sonoma Technology, Inc, Petaluma, California, USA; 5 Swiss Tropical and Public Health Institute, Basel and University of Basel, Switzerland; 6 United Health Group, City of Hope Hospital Medical Center, Los Angeles, California, USA

**Keywords:** air pollution, asthma, child, epidemiology, vehicular traffic

## Abstract

**Background:**

Traffic-related air pollution has been associated with adverse cardiorespiratory effects, including increased asthma prevalence. However, there has been little study of effects of traffic exposure at school on new-onset asthma.

**Objectives:**

We evaluated the relationship of new-onset asthma with traffic-related pollution near homes and schools.

**Methods:**

Parent-reported physician diagnosis of new-onset asthma (*n* = 120) was identified during 3 years of follow-up of a cohort of 2,497 kindergarten and first-grade children who were asthma- and wheezing-free at study entry into the Southern California Children’s Health Study. We assessed traffic-related pollution exposure based on a line source dispersion model of traffic volume, distance from home and school, and local meteorology. Regional ambient ozone, nitrogen dioxide (NO_2_), and particulate matter were measured continuously at one central site monitor in each of 13 study communities. Hazard ratios (HRs) for new-onset asthma were scaled to the range of ambient central site pollutants and to the residential interquartile range for each traffic exposure metric.

**Results:**

Asthma risk increased with modeled traffic-related pollution exposure from roadways near homes [HR 1.51; 95% confidence interval (CI), 1.25–1.82] and near schools (HR 1.45; 95% CI, 1.06–1.98). Ambient NO_2_ measured at a central site in each community was also associated with increased risk (HR 2.18; 95% CI, 1.18–4.01). In models with both NO_2_ and modeled traffic exposures, there were independent associations of asthma with traffic-related pollution at school and home, whereas the estimate for NO_2_ was attenuated (HR 1.37; 95% CI, 0.69–2.71).

**Conclusions:**

Traffic-related pollution exposure at school and homes may both contribute to the development of asthma.

The role of air pollution in the development of new-onset asthma remains controversial, and the contribution of this environmental risk factor to the pandemic remains unclear (Eder et al. 2006; Sarnat and Holguin 2007). Although increasing evidence indicates that living near heavy traffic is associated with increased rates of asthma, some well-designed studies have found only weak or no associations (Heinrich and Wichmann 2004; Oftedal et al. 2009).

These inconsistencies may reflect incomplete assessment of exposures to traffic in the microenvironments in which children spend most of their time. Exposure at locations other than home, especially at school where children spend a large proportion of waking hours and may engage in physical activity that would increase the ventilation rate and dose of inhaled pollutants, may have strong influences on occurrence of asthma. However, few studies have examined the effect of traffic-related pollution at schools on asthma rates among children. Two cross-sectional Dutch studies that examined this question reported higher rates of respiratory symptoms among children in schools near roadways with heavy traffic, especially truck traffic (Janssen et al. 2003; van Vliet et al. 1997). A northern California study found that schools downwind from busy freeways had higher concentrations of oxides of nitrogen (NO_x_) and higher asthma prevalence rates (Kim et al. 2004). However, in a subsequent analysis of these same data, the effect of school exposure was attenuated and no longer significant after adjusting for modeled residential traffic-related exposure (Kim et al. 2008).

Nitrogen dioxide (NO_2_) is routinely measured at regulatory monitoring stations. However, these measurements reflect both background regional concentrations and local sources near the stations. Some studies have assessed local exposure to traffic-related pollution using measured NO_2_ (or NO_x_) as surrogate for the complex mixture of traffic-related pollutants that occurs in close proximity to roadways (Brauer et al. 2007; Gauderman et al. 2005; Jerrett et al. 2008; Kim et al. 2004; Kramer et al. 2000; Nordling et al. 2008). NO_2_ can feasibly be measured at a large number of locations, and it has been widely used as a proxy for the mixture of traffic-related pollutants that vary markedly depending on distance from roadways, season, wind speed, and wind direction. However, the mixture of pollutants in close proximity to roadways includes transition metals and organic aerosols, which are more plausible causes of asthma than NO_2_ (Li et al. 2003).

The Southern California Children’s Health Study (CHS) was designed to investigate the chronic effects of air pollution on respiratory health. Previous analyses have found associations of asthma with residential distance to major roads and modeled and measured pollutant markers for intracommunity variation in exposure to traffic (Gauderman et al. 2005; Jerrett et al. 2008; McConnell et al. 2006; Salam et al. 2007b) and with regional pollutants in susceptible children (Islam et al. 2008; McConnell et al. 2002). In the current analysis, a prospective, longitudinal evaluation of new cases of asthma has allowed assessment of exposure before the development of asthma in an ongoing cohort of school children recruited in kindergarten and first grade, an age at which physician-diagnosed asthma becomes reliable and valid (Martinez et al. 1995). Exposure to traffic-related pollutants has been estimated at participating schools and at residences. In addition, NO_2_, ozone (O_3_), and particulate matter < 10 μm (PM_10_) and < 2.5 μm in aerodynamic diameter (PM_2.5_) have been measured continuously during the lifetime of most study participants at a location in each community representative of exposure in the neighborhoods where children live (Kunzli et al. 2003; McConnell et al. 2006). This design allowed examination of the joint effects of traffic-related pollution exposure at school and home and of regional measured pollution at community monitors. We hypothesized that exposure at school and home both contribute to the risk of new-onset asthma.

## Methods

### Study population

Characteristics of this cohort have been described previously (McConnell et al. 2006). Briefly, 5,349 children attending kindergarten and first grade were enrolled into a new CHS cohort during the 2002–2003 school year from 45 schools in 13 communities. Communities were selected to represent the range and mixture of regional particulate pollutants, NO_2_, and O_3_ in southern California. Parents provided informed consent and completed a baseline and yearly questionnaire with information about demographic characteristics, respiratory illness, and risk factors for asthma at study entry. Children with a history of physician-diagnosed asthma at study entry (*n* = 715, 13.4%) were excluded from this follow-up, because the focus was on risks for incident asthma. To ensure that the study population was free of any previously undiagnosed asthma, we then also excluded children with a history of wheeze (*n* = 868, 16.2%) and additional children with missing information about wheeze, or missing or a “don’t know” answer about asthma (*n* = 394, 7.4%). These exclusions resulted in a total of 3,372 children classified as disease free at baseline. We excluded another 340 children with no residential traffic information because home address could not be geocoded [disproportionately from a single community, as described in the Supplemental Material (doi:10.1289/ehp.0901232)] and another 535 children who did not participate in any of the annual follow-up questionnaires. The final sample included 2,497 children.

### New-onset asthma and covariates

Children with physician-diagnosed asthma reported on a yearly questionnaire during 3 years of follow-up were defined to have new-onset asthma. Children missing questionnaires in any year continued to contribute person-time to the models until they answered yes (at which time they were censored) or were lost to follow-up. The date of new onset of asthma could not be precisely defined. Therefore, the date of onset was assigned to the midpoint of the interval between the date of the questionnaire when asthma diagnosis was first reported and the date of the previous questionnaire reporting asthma status, and this date was used for estimating follow-up time in all statistical analyses. Sociodemographic characteristics, exposure to cigarette and wildfire smoke, health insurance, housing characteristics, history of allergy, and parental asthma were assessed by questionnaire [see Supplemental Material (doi:10.1289/ehp.0901232) for details].

### Community ambient air pollution measurements

Ambient levels of O_3_, NO_2_, PM_10_, and PM_2.5_ were measured continuously during follow-up at a central site monitor in each community, as described in the Supplemental Material (doi:10.1289/ehp.0901232) and in previous reports (Peters et al. 1999). Because there is marked diurnal variation in O_3_ and exposure occurs largely between 1000 and 1800 hours, this average was used in all analyses. Average temperature and relative humidity were also obtained during follow-up from measurements at the monitoring stations.

### Local traffic-related pollutant exposure

Exposure to traffic-related pollution was estimated using methods described previously (McConnell et al. 2006), and several metrics of local traffic related exposure were compiled. Participant residence and school addresses were geocoded, and we estimated distances to the nearest freeway or other highways or arterial roads and traffic density within 150 m of each child’s residence and school. Concentrations of pollutants from local vehicle emissions at homes and schools were estimated separately from freeway and nonfreeway sources, using a line source dispersion model of the incremental contribution by these nearby sources to oxides of nitrogen above the regional background levels (Benson 1989). The modeled annual concentration estimates (parts per billion) were based on distance to roadways, vehicle counts, vehicle NO_x_ emission rates, wind speed and direction, and height of the mixing layer in each community. However, these modeled exposures reflect the mixture of multiple pollutants from nearby traffic, and the high correlation of pollutants in the mixture precludes identifying the effect of any specific pollutant in the mixture, as described in the Supplemental Material (doi:10.1289/ehp.0901232). For brevity, these modeled exposures will be referred to as traffic-related pollutants (TRP). An estimate of combined school and home exposure to traffic was made by weighting the estimates by approximate time at school (35 hr weekly, or 21%) and assigning the remaining 79% of time to the home. Health effect estimates based on models in which the contribution of school to the combined exposure was reduced to 16% to account for a lack of school exposure during summer recess were very similar (data not shown).

### Statistical methods

We fitted a multilevel Cox proportional hazards model that allows for assessment of residual variation in time to asthma onset and also for clustering of children around schools and communities (Ma et al. 2003). Letting *u_i_* and *u_ij_* denote community- and school (within community)-level random effects, with *u_ij_* assumed to be positive and independent conditional on *u_i_*, we fit the following model:


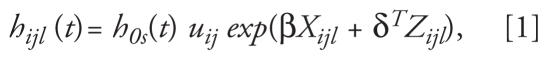


where, for the *l*th subject in the *i*th community, and *j*th school, *h_ijl_*(*t*) is the hazard function at age *t; h_0s_*(*t*) is the baseline hazard function in stratum *s* (defined by age at study entry and sex), *X_ijl_* is a traffic pollution exposure metric, and *Z_ijl_* are other individual covariates, such as secondhand smoke exposure, pets in the home, and other possible confounders. All models included race/ethnicity.

The model allowed for joint evaluation of the effects of exposure to traffic-related pollutants at homes and at schools and to ambient pollutants measured at community central sites, with effects scaled to the interquartile range (IQR) for each metric of residential exposure (e.g., for TRP from the line source dispersion model) and to the total range across the 13 communities, respectively. Traffic exposure at homes and school were correlated. Therefore, in models including both exposures, home traffic estimates were deviated from the corresponding school metric to minimize the chance of collinearity and allow for valid independent effect estimates (for example, *r*-square for school with deviated home TRP were < 0.38 for freeway, nonfreeway, and total TRP). Although the school-specific estimates of exposure were of primary interest, models including the average residential exposure corresponding to each school (and residential exposures deviated from this average) were also fitted [see Supplemental Material (doi:10.1289/ehp.0901232) for details]. Potential confounders were evaluated one at a time, based on whether the estimates for the pollutant associations changed by > 10%. We assessed heterogeneity of traffic pollution effects by level of community central site regional pollutant measurements by comparing nested models using a partial likelihood ratio test with and without interaction terms. We examined any potential nonlinearity in the exposure–response relationship using cubic spline terms, piecewise polynomials joined smoothly at a number of break points (Hastie and Tibshirani 1990), for the exposure terms and comparing the nested models using a partial likelihood ratio test. All analyses were conducted using software designed to run within R software (R Development Core Team, Vienna, Austria) for implementing random effects Cox proportional hazards models (Krewski et al. 2009; Ma et al. 2003). All hypotheses were tested assuming a 0.05 significance level and a two-sided alternative hypothesis.

## Results

Most of the 2,497 disease-free children included in this analysis were ≤ 6 years of age (range, 4.8–9.0 years) at study entry (Table 1). There were 120 new cases of asthma, resulting in an incidence rate of approximately 18.7 cases per 1,000 person-years (based on 6,434 person-years of follow-up). Rates did not differ by age at study entry or sex. About half the children were Hispanic whites, and they had the lowest rates of asthma (16.8/1,000 person years), whereas African Americans had the highest rate (33.9/1,000 person-years), but this was based on only six cases. Substantially higher rates were also observed for children with history of allergy, parental history of asthma, and maternal smoking during pregnancy.

Both residential and school TRP metrics had a wide range with skewed distributions (Table 2). For example, the mean and median for nonfreeway residential TRP were 7.3 and 6.1 ppb, respectively, and the IQR was 8.0 ppb, with a total range from 0.08 to a maximum of 55.1 ppb. A few homes and schools had very low freeway TRP (largely in a single community that has no freeways). There was ≥ 3-fold variation in the multiyear average NO_2_, PM_10_, and PM_2.5_ from the lowest to highest pollution community for measurements at the central site monitors. O_3_ levels varied by 2-fold.

The associations of new-onset asthma were strongest with nonfreeway TRP. The hazard ratio (HR) at homes was 1.51; 95% confidence interval (CI), 1.25–1.81; *p* < 0.001 (Table 3). The HR for exposure at schools was almost as large as for residential exposure (HR = 1.45; 95% CI, 1.06–1.98), and the combined average exposure weighted for time at school and home had a slightly stronger association (HR = 1.61; 95% CI, 1.29–2.00). These associations were not confounded by any of the covariates in Table 1 or by community relative humidity, which we have previously found to confound TRP exposure (Jerrett et al. 2008). There also were no significant interactions of either residential or school nonfreeway TRP with sex, allergy, or parental history of asthma, which we have previously shown modified effects of traffic exposure (McConnell et al. 2006). Similar effect sizes were observed in sensitivity analyses restricted to lifetime residents at the same address, analyses excluding children diagnosed in the first year of follow-up and excluding children who changed schools during the follow-up period. There was little evidence of nonlinearity in the exposure–response relationship based on sensitivity analyses comparing the fit of a smoothed cubic spline model of asthma with a linear model (*p*-value > 0.80) for the partial likelihood ratio test for models with 3 and 5 knots compared with the linear model. Weaker associations were observed with total TRP at homes (HR = 1.32; 95% CI, 1.08–1.61) and school (HR = 1.20; 95% CI, 0.91–1.58). There were weaker and nonsignificant associations with TRP modeled from freeways. Nonfreeway TRP was moderately to strongly correlated with freeway TRP at homes (*R* = 0.64) and at schools [*R* = 0.70; Supplemental Material, Table E-1, (doi:10.1289/ehp.0901232)]. However, in models co-adjusted for freeway and nonfreeway TRP, the nonfreeway effect estimates for school and home were similar to the unadjusted estimates.

Of the regional community central-site pollutants, NO_2_ was associated with more than double the risk of new-onset asthma (HR = 2.17; 95% CI, 1.18–4.00; *p* = 0.01) over the range of exposure (23.6 ppb) across the 13 study communities (Table 4). In a model with NO_2_, school and residential nonfreeway TRP exposure, the estimate for NO_2_ was attenuated (HR = 1.37; 95% CI, 0.69–2.71; Table 5). However, the adjusted associations of asthma with nonfreeway TRP at homes (HR = 1.46) and schools (HR = 1.45) in Table 5 were very similar to the unadjusted effect estimates (HR = 1.51 and 1.45, respectively, shown in Table 3). In sensitivity analyses, a similar pattern of attenuation of regional NO_2_ effect estimates was observed in models weighted for time at school and home (results not shown), and in models adjusted for total (freeway plus nonfreeway) TRP exposure at school and home [Supplemental Material, Table E-2 (doi:10.1289/ehp.0901232)]. There were neither statistically significant interactions of central site pollutants with residential TRP nor any consistent patterns suggestive of different effects in high and low regional pollutant communities (results not shown). We have previously shown that O_3_ modified the association of team sports participation with new-onset asthma in an older cohort (McConnell et al. 2002), but there was neither a main effect of sports nor confounding or effect modification of effects of residential TRP or ambient central monitor pollutant exposure.

Distance to freeways and other major roads and traffic volume on those roads have been used in other studies as independent predictors of traffic exposure, so we examined the distribution [Supplemental Material, Table E-3 (doi:10.1289/ehp.0901232)] and correlations of these metrics (Supplemental Material, Table E-1) and the association at school and home with asthma (Supplemental Material, Tables E-4 and E-5). In general, these exposure metrics were weakly to moderately correlated with the TRP metrics but were not consistently associated with asthma in our data set.

## Discussion

The study is unique in its prospective assessment of the relationship of new-onset childhood asthma to community regional air pollution and near-source traffic-related exposure at home and in a large number of schools. The results indicate that associations of asthma with traffic-related pollution from nearby sources at schools were independent of estimated effects of exposures at homes, and these effects were of similar size over a range of exposure common in Southern California. Evidence of a school effect comparable with that associated with the much larger amount of time spent at home could potentially be explained by physical education and other exercise at school that may increase ventilation rate and dose of pollutants to the lungs and thereby increase the risk associated with exposure. Traffic-related pollutant levels may also be considerably higher during the morning hours, when children are arriving at school, especially during temperature inversions that occur largely in the winter months when children are attending school (Kim et al. 2002; Ning et al. 2007). It is possible that school exposure may have reflected time spent in other locations in the child’s neighborhood rather than school-specific exposure, and this possibility merits further investigation. However, time at school during the school year accounts for more than one-third of all waking hours, and in this age group it is likely that most of the child’s remaining nonschool time was spent at home (Xue et al. 2004). The increased risk associated with TRP could not be explained by confounders commonly associated with asthma or by ambient NO_2_ or other currently regulated regional pollutants measured at the central-site monitoring stations. These results strengthen an emerging body of evidence from both toxicologic and epidemiologic studies that air pollutants from nearby traffic contribute to the development of asthma (Salam et al. 2008).

NO_2_ measured at community monitoring stations has been associated with wheeze prevalence in an older CHS cohort (Peters et al. 1999) and with increased risk of asthma incidence in a Japanese cohort (Shima et al. 2002). We have recently reported associations of both incident and prevalent asthma with ambient residential NO_2_ measured outside homes of a relatively small sample of children from another CHS cohort in many of the same communities, but NO_2_ in that study was a marker for intracommunity variation in the mixture of TRPs (Gauderman et al. 2005; Jerrett et al. 2008). Other studies also indicate that associations of respiratory disease with intracommunity variation in NO_2_ reflect the effects of other TRP rather than NO_2_ (Kramer et al. 2000). A study that measured NO_2_ and NO_x_ in central neighborhood locations found that associations with asthma were attenuated by adjustment for modeled residential traffic exposure (Kim et al. 2004, 2008). In the current study, the attenuated association between asthma and NO_2_ continuously measured at the community monitor in models with adjustment for TRP also suggests that NO_2_ was not causally related to asthma. However, interpretation of this result is not clear because exposure measured at the community monitor may have misclassified exposures of children in parts of the community with significant local sources of NO_2_. We did not find evidence for main effects of regional O_3_ and PM with asthma, consistent with results from previous analyses in older CHS cohorts, although we have previously shown that O_3_ modified the effect of outdoor exercise in a genetically susceptible subgroup (Islam et al. 2009; McConnell et al. 2002).

Associations with asthma were significant for TRP exposure estimates modeled from local nonfreeway roadway proximity, traffic volume, and meteorology. There was little evidence for an effect of major roadway proximity alone, for traffic density, or for pollution from freeways. The absence of a freeway TRP effect suggests that causal pollutants may be highly reactive, resulting in steep spatial gradients from freeway sources and little exposure at the longer distances of homes and schools to freeways compared with distance to other major roads [Supplemental Material, Table E-3 (doi:10.1289/ehp.0901232)]. Several previous studies have found that the largest gradients in traffic-related pollutants occur within 150 m from roadways (Gilbert et al. 2003; Zhu et al. 2002), and variability has often been best explained by traffic volume within 300 m (Briggs et al. 2000; Gilbert et al. 2003; Ross et al. 2006). High-volume traffic on secondary roads may also produce pollutants resulting from frequent stops and accelerations, which have been found to be associated with asthma symptoms (Ryan et al. 2005). However, in an older cohort from some of these same communities, we observed associations of freeway-modeled exposures and residential freeway proximity with respiratory outcomes (Gauderman et al. 2005, 2007). Different traffic metric associations with respiratory health may have been due to variation in the distribution of freeway and nonfreeway traffic around homes in the different cohorts and the relatively small number of children living near freeways (< 7% within 150 m in the current cohort). It is also possible that the accuracy of exposure models for the freeway and nonfreeway sources varied in the two cohorts or that the greater mobility of older children resulted in health effects associated with spending time in areas such as parks in close proximity to freeways near homes.

An important distinction between the TRP and simpler traffic metrics is the inclusion of meteorology (average annual wind speed and direction and height of the mixing layer) in addition to proximity and volume. Traffic density alone [Supplemental Material, Table E-4 (doi:10.1289/ehp.0901232)] and proximity to a major roadway (Supplemental Material, Tables E-4 and E-5) had weaker positive associations with incident asthma that were not statistically significant. Therefore, inconsistent findings in some of the (largely cross-sectional) studies that have evaluated associations with asthma (Heinrich and Wichmann 2004) may reflect the failure to account for the impact of meteorology on exposure. We have recently measured NO_x_ at > 900 locations in these communities. Approximately two thirds of the within-community variability in NO_x_ was explained by TRP, a substantially higher amount of variation than was explained by any other traffic metric (unpublished data). We have also previously observed that both TRP and residential traffic proximity were associated with increased asthma prevalence in this cohort (McConnell et al. 2006).

The prospective design is a strength of this study. In other prospective studies of birth cohorts in Sweden and the Netherlands, modeled traffic-related pollutants, including NO_x_ and/or PM_2.5_, were associated with incidence of wheeze and atopy (Nordling et al. 2008) and doctor-diagnosed asthma (Brauer et al. 2007) in children < 5 years of age, when the diagnosis of asthma may be difficult to distinguish from transient wheeze not predictive of asthma. As these cohorts mature, the relationship to asthma is likely to become more clear. For example, associations between PM_2.5_ light absorption, roadway proximity, and asthmatic bronchitis and atopy were found in a study of two German birth cohorts that followed some children to 6 years of age (Morgenstern et al. 2008). Associations with allergic symptoms in these cohorts were less robust at an earlier age (Gehring et al. 2002). Other recent results from prospective studies of birth cohorts (Gehring et al. 2009) and of children (Shima et al. 2002) followed to older ages have generally shown positive associations between markers of residential TRP and asthma, although a recent Norwegian study found no association with modeled exposure at the birth address of a large cohort of children (Oftedal et al. 2009). Genetic studies examining pathways likely to mediate effects of air pollution also strengthen the causal inference that traffic-related pollutants may cause asthma. We have shown elsewhere that the risk of asthma associated with traffic exposure and with traffic-related ambient PM was modified in a predictable way by functional gene variants in pathways associated with asthma, including inflammation and airway remodeling (Salam et al. 2007a) and oxidative stress (Islam et al. 2008; Salam et al. 2007b). In another analysis of the data from this cohort, we have shown that the estimated effect of TRP was modified by socioeconomic status and psychosocial stress (Shankardass et al. 2009).

There are some limitations to these data. Our conservative estimates of TRP for the center of school buildings are likely to underestimate the exposure of students in buildings closer to the roadways, especially during dropoff and pickup of students at the beginning and end of the school day. The impact of this underestimate of exposure on the assessment of health effects is not clear, although in general it might be expected that this exposure misclassification would be nondifferential with respect to asthma and would result in underestimation of the effect of traffic exposure. Other limitations include the relatively short (3-year) period of follow-up and the retrospective questionnaire assessment of early-life risk factors, as this was not a pregnancy cohort. Our cohort excluded children who left the community before school entry and who had asthma or wheeze at study entry. However, our cohort allowed us to examine risk factors in school children, an age during which there has been little study of new-onset asthma.

Asthma is a clinical syndrome with no sensitive and specific diagnostic test available to confirm clinical assessments. Because of the clinical nature of the assessment, the reported physician diagnosis of asthma we used has been recommended and widely used as a method to classify asthma status in epidemiologic studies, although this approach has limitations (Asher et al. 1995; Burr 1992). If misclassification of true asthma based on this approach were random with respect to exposure to air pollution, then the observed associations would be likely to have underestimated the effect of exposure. Bias could explain our results if asthma misclassification were related also to exposure. This could occur if exposure were associated with access to care and differences in practice among physicians that have the potential to influence asthma diagnosis (Samet 1987). However, adjustment for factors that mediate access to care including family income, parental education, and having medical insurance did not alter our results, indicating that confounding due to differential access to care was unlikely to explain these findings. The validity of questionnaire-reported physician diagnosis has also been established in a subset of CHS participants (Salam et al. 2007a). In addition, any child with a history of wheezing at study entry was excluded from the analysis, so it is unlikely that the exacerbation of undiagnosed prevalent asthma by traffic explained these associations. Loss to follow-up could also potentially result in bias because of selection. Sociodemographic characteristics varied between the analysis cohort and children not contributing to the analysis because of loss to follow-up or missing or poorly matched geocodes for addresses precluding estimation of residential exposure [Supplemental Material, Table E-6 (doi:10.1289/ehp.0901232)]. Hispanic children were less likely than non-Hispanic white children to be included in the follow-up analysis, as were children without insurance and with lower parental education and income. However, adjusting for these factors had little impact on the pattern of observed effects. In addition, there was little difference between exposure to nonfreeway TRP in the study sample (7.3 ppb) and in the group not included because of loss to follow-up (7.6 ppb). Therefore, it is unlikely that selection bias due to loss to follow-up explained our results.

## Conclusions

An estimated 6.2 million children have asthma in the United States, making it the most common chronic disease in childhood (Moorman et al. 2007), and rates have increased markedly in developed countries over the past several decades (Braman 2006). Morbidity results in impaired quality of life for the affected child and other family members, in increased use of health services, and in school absences that have large social and economic costs (Wang et al. 2005). Our results indicate that children exposed to higher levels of traffic-related air pollution at school and home are at increased risk of developing asthma. Almost 10% of public schools in California are located within 150 m of roadways with > 25,000 vehicles daily (Green et al. 2004). Students in urban areas in eastern U.S. cities are even more likely than children in Los Angeles to attend schools near major highways (Appatova et al. 2008). Therefore, exposure to TRP is potentially an important public health problem affecting large populations of children. Planning transportation and other urban development to limit population exposure to traffic exhaust, as well as more effective control of vehicular emissions, may result in substantial long-term public health benefits.

## Figures and Tables

**Table 1:** Characteristics of participants at study entry and incidence of asthma on follow-up.

Characteristic	*n*^a^ (%)	Cases	IR^b^ (95% CI)
Age at entry (years)			
< 6	496 (20)	22	17.0 (11.2–25.9)
6	1,178 (47)	58	19.0 (14.7–24.6)
> 6	823 (33)	40	18.9 (13.9–25.8)

Race/ethnicity			
Hispanic white	1,380 (55)	59	16.8 (13.0–21.7)
Non-Hispanic white	905 (36)	45	18.9 (14.0–25.2)
African American	77 (3.1)	6	33.9 (15.2–75.4)
Asian	97 (3.9)	8	30.8 (15.4–61.5)
Other/unknown	38 (1.5)	2	22.7 (5.7–90.8)

Sex			
Female	1,307 (52)	62	18.2 (14.2–23.3)
Male	1,190 (48)	58	19.2 (14.8–24.8)

History of allergy			
Yes	764 (34)	56	28.7 (22.1–37.4)
No	1,508 (66)	50	12.8 (6.7–24.4)

Play team sport			
Yes	918 (39)	47	19.7 (14.8–26.2)
No	1,463 (61)	67	17.9 (9.2–34.5)

Parental history of asthma			
Yes	389 (17)	31	31.3 (22.0–44.6)
No	1,899 (83)	76	15.4 (7.2–33.3)

Maternal smoking during pregnancy			
Yes	156 (6.3)	11	27.9 (15.5–50.5)
No	2,308 (93.7)	107	18.0 (5.4–60.3)

Secondhand smoke			
Yes	164 (6.7)	7	17.0 (8.1–35.6)
No	2,281 (93.3)	110	18.7 (4.1–84.0)

Mildew			
Yes	532 (22)	29	21.1 (14.7–30.4)
No	1,911 (78)	85	17.3 (7.9–37.9)

Pests in home			
Yes	1,603 (67)	75	18.1 (14.4–22.7)
No	777 (33)	36	18.2 (9.7–33.9)

Dogs in home			
Yes	730 (30)	30	15.7 (11.0–22.4)
No	1,706 (70)	86	19.7 (9.1–42.8)

Cats in home			
Yes	462 (19)	17	14.0 (8.7–22.5)
No	1,972 (81)	98	19.4 (7.2–52.2)

Indoor NO_2_ source			
Yes	1,799 (73)	88	18.8 (15.3–23.2)
No	675 (27)	31	18.3 (9.9–33.9)

Wildfire exposure^c^			
Yes	251 (12)	16	24.6 (15.1–40.1)
No	1,764 (88)	79	17.3 (6.2–48.2)

Health insurance			
Yes	2,135 (88)	106	19.2 (15.9–23.3)
No	300 (12)	10	13.1 (5.7–30.3)

Household income (US$)			
≤ 14,999	329 (14)	16	19.5 (11.9–31.8)
15,000–49,999	714 (31)	38	21.1 (15.4–29.0)
> 50,000	1,292 (55)	57	16.6 (12.8–21.6)

Parental education			
Less than high school	508 (21)	23	17.9 (11.9–26.9)
At least high school	452 (19)	20	17.5 (11.3–27.1)
Some college	850 (36)	47	20.0 (14.9–26.9)
College and above	560 (24)	24	13.9 (9.1–21.3)
Total	2,497	120	18.7 (15.6–22.3)

a Numbers may not add up to 2,497 in some subgroups because of missing data.

b Crude incidence (IR) rate per 1,000 person-years.

c Occurred in 2003 during first year of follow-up.

**Table 2:** Distribution of annual average residential traffic-modeled pollution and of community central site measurements.

	Mean	Median	IQR	Minimum	Maximum	Range
Residential traffic (ppb)						
Nonfreeway TRP	7.3	6.1	8	0.08	55.1	55
Freeway TRP	11.1	7.3	13	< 0.0001	134.5	134.5
Total TRP	18.4	14.6	20.9	0.23	144.1	143.9

School traffic (ppb)						
Nonfreeway TRP	6.1	5.9	5.9	0.3	19.7	19.4
Freeway TRP	10.9	8.2	15.2	< 0.0001	39.9	39.9
Total TRP	17	11.9	18.5	0.7	51.4	50.7

Central-site measurements						
NO_2_ (ppb)	20.4	21.8	12.8	8.7	32.3	23.6
PM_10_ (μg/m^3^)	35.5	34	11.7	17.6	61.5	43.9
PM_2.5_ (μg/m^3^)	13.9	15.1	9.7	6.3	23.7	17.4
1000–1800 hours O_3_ (ppb)	44.6	43.6	11.1	29.5	59.8	30.3

**Table 3:** Association between new-onset asthma and modeled exposure at home and school.

Traffic-related exposure^a^	Home HR^b^ (95% CI)	School HR^b^ (95% CI)	Combined^c^ HR^b^ (95% CI)
Nonfreeway TRP	1.51 (1.25–1.81)*	1.45 (1.06–1.98)	1.61 (1.29–2.00)*
Freeway TRP	1.12 (0.95–1.31)	1.08 (0.86–1.34)	1.12 (0.94–1.35)
Total TRP	1.32 (1.08–1.61)**	1.20 (0.91–1.58)	1.34 (1.07–1.68)

a Scaled to the IQR at homes for each metric (from Table 2).

b HR (95% CI, adjusted for race/ethnicity and for baseline hazards strata of age at study entry and sex) with random effects for community and school.

c Combined weighted for time at home and school.

* *p* < 0.001.

** *p* < 0.01.

**Table 4:** Association of new-onset asthma with community central site pollutant measurements.

Pollutant	HR^a^ (95% CI)
NO_2_	2.17 (1.18–4.00)
PM_10_	1.35 (0.64–2.85)
PM_2.5_	1.66 (0.91–3.05)
O_3_	0.76 (0.38–1.54)

a HR (95% CI) across the range of exposure in the 13 communities (23.6 ppb for NO_2_, 43.9 μg/m^3^ PM_10_, 17.4 μg/m^3^ PM_2.5_, and 30.3 ppb for 1000–1800 hours O_3_), adjusted for race/ethnicity and for baseline hazards strata of age at study entry and sex with random effects for community and school.

**Table 5:** Mutually adjusted associations of new-onset asthma with community central site pollutant measurements and nonfreeway TRP at home and school.^a^

Central site pollutant	HR^b^ (95% CI) for ambient pollutant, adjusted for TRP at home and school	HR^b^ (95% CI) for home TRP, adjusted for school TRP and ambient pollutant	HR^b^ (95% CI) for school TRP, adjusted for home TRP and ambient pollutant
NO_2_	1.37 (0.69–2.71)	1.46 (1.16–1.84)*	1.45 (1.03–2.06)
PM_10_	1.40 (0.62–3.17)	1.46 (1.16–1.85)*	1.53 (1.10–2.12)*
PM_2.5_	1.30 (0.66–2.56)	1.48 (1.19–1.85)*	1.49 (1.07–2.08)
O_3_	1.01 (0.49–2.11)	1.50 (1.20–1.86)*	1.54 (1.10–2.14)*

a Mutually adjusted across each row (i.e., effect of each community pollutant was examined separately in a model including both home and school TRP).

b HR (95% CI) for central-site pollutants scaled across the range of exposure in the 13 communities (23.6 ppb for NO_2_, 43.9 μg/m^3^ PM_10_, 17.4 μg/m^3^ PM_2.5_, and 30.3 ppb for 1000–1800 hours O_3_); household nonfreeway TRP was deviated from school, scaled to the IQR for home exposure (8 ppb from Table 2).

* *p* < 0.01.
